# Coombs-Negative Hemolytic Anemia and Thrombocytopenia Associated With Sarcoidosis Confined to the Spleen and Bone Marrow: A Case Report

**DOI:** 10.7759/cureus.105385

**Published:** 2026-03-17

**Authors:** Yoshiki Uemura, Kazuto Togitani, Junko Nakashima

**Affiliations:** 1 Department of Hematology, Chikamori Hospital, Kochi, JPN; 2 Department of Pathology, Chikamori Hospital, Kochi, JPN

**Keywords:** coombs-negative, hemolytic anemia, soluble interleukin-2 receptor, splenectomy, splenic sarcoidosis

## Abstract

Sarcoidosis is a systemic granulomatous disease that typically involves the lungs and lymphatic system. Isolated splenic sarcoidosis with bone marrow involvement is exceedingly rare, and hemolytic anemia associated with sarcoidosis is usually Coombs-positive. We report a case of a 61-year-old man who presented with massive splenomegaly, Coombs-negative hemolytic anemia, and thrombocytopenia. Serum angiotensin-converting enzyme (ACE) and soluble interleukin-2 receptor (sIL-2R) levels were markedly elevated, strongly suggesting malignant lymphoma. Bone marrow biopsy revealed non-caseating granulomas without evidence of malignancy, and 18F-FDG PET/CT demonstrated intense uptake in the spleen with diffuse uptake in the bone marrow. Treatment with prednisolone and a thrombopoietin receptor agonist improved thrombocytopenia, enabling splenectomy. Histopathological examination of the spleen confirmed sarcoidosis. This case highlights the diagnostic challenge of distinguishing sarcoidosis from lymphoma in patients with isolated splenomegaly and markedly elevated sIL-2R levels and demonstrates that sarcoidosis confined to the spleen and bone marrow can present with Coombs-negative hemolytic anemia and thrombocytopenia, with splenectomy playing a crucial role in definitive diagnosis and management.

## Introduction

Sarcoidosis is a systemic granulomatous disease of unknown etiology, most commonly affecting the lungs and lymphatic system [1]. Although extrapulmonary manifestations are not uncommon, isolated splenic sarcoidosis in the absence of pulmonary involvement is exceedingly rare. When splenomegaly is accompanied by elevated serum angiotensin-converting enzyme (ACE) levels, sarcoidosis may be suspected. However, markedly increased levels of soluble interleukin-2 receptor (sIL-2R) often raise concern for malignant lymphoma, creating a diagnostic challenge [2].

Here, we report a rare case of isolated splenic sarcoidosis with bone marrow involvement, presenting with massive splenomegaly, Coombs-negative hemolytic anemia, and thrombocytopenia. Coombs-negative hemolytic anemia, defined as hemolysis in the absence of detectable anti-erythrocyte antibodies on the direct antiglobulin test, is a diagnostically challenging condition. Hemolytic anemia in sarcoidosis is an uncommon finding, and most reported cases are Coombs-positive [3]. To our knowledge, only one prior case has described Coombs-negative hemolytic anemia in the context of isolated splenic sarcoidosis [4], underscoring the uniqueness of the present case. Despite the elevated ACE level suggestive of sarcoidosis, the markedly increased sIL-2R level initially led to a clinical suspicion of malignant lymphoma. A definitive diagnosis was ultimately achieved by histopathological examination of the resected spleen following splenectomy, which also resulted in resolution of the hematologic abnormalities. This case emphasizes the diagnostic complexities and therapeutic considerations in patients presenting with isolated splenomegaly and unexplained cytopenias. Written informed consent for publication was obtained from the patient.

## Case presentation

A 61-year-old man presented with complaints of fatigue and low-grade fever. Physical examination revealed marked splenomegaly without hepatomegaly or palpable superficial lymphadenopathy. Laboratory findings showed normocytic anemia (hemoglobin, 6.4 g/dL; reference range, 11.6-14.8 g/dL) and thrombocytopenia (platelet count, 6.2 × 10⁴/μL; reference range, 15.8-34.8 × 10⁴/μL).

Hemolytic anemia was diagnosed based on elevated lactate dehydrogenase (LDH), indirect hyperbilirubinemia, decreased haptoglobin, and peripheral blood smear findings showing polychromasia and anisocytosis without schistocytes or abnormal lymphoid cells. Serological testing for hepatitis C virus (HCV) was negative, thereby excluding hepatitis C-associated splenic marginal zone lymphoma.

**Figure 1:**
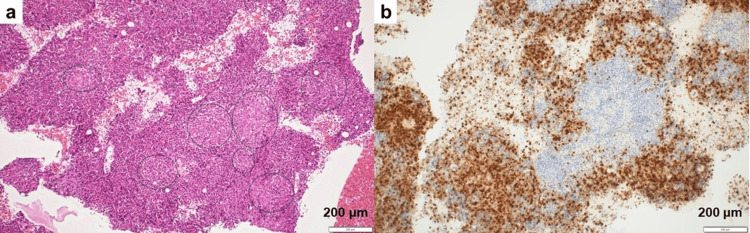
Histopathological findings of the bone marrow clot section (a) Hematoxylin and eosin staining (×100): The marrow shows marked hypercellularity with nearly complete absence of adipocytes. No dysplasia is observed in the erythroid, myeloid, or megakaryocytic lineages. Numerous non-caseating granulomas with focal necrosis are present. The area of interest is highlighted by a black circle. There is dense infiltration of lymphocytes without atypia, and no findings suggestive of malignant lymphoma are identified. (b) Myeloperoxidase staining (×100): Granulocytic lineage cells are displaced by non-caseating granulomas.

Bone marrow biopsy showed no evidence of lymphoma cell infiltration. The marrow was normocellular with preserved trilineage hematopoiesis, without dysplastic changes, blast increase, or clonal hematologic abnormalities. Numerous non-caseating epithelioid cell granulomas were observed (Figure 1), consistent with sarcoidosis, confirming granulomatous disease.

To exclude infectious granulomatous diseases, additional histochemical analyses were performed using the paraffin-embedded splenic tissue. Ziehl-Neelsen staining for acid-fast bacilli and Grocott and periodic acid-Schiff (PAS) staining for fungal organisms were all negative.

Serum angiotensin-converting enzyme (ACE) was also elevated at 45.9 U/L (reference range, 8.3-21.4 U/L). The soluble interleukin-2 receptor (sIL-2R) level was markedly elevated at 26,600 U/mL (reference range, 157-474 U/mL), which prompted consideration of an underlying lymphoproliferative disorder involving the spleen. Despite the histological findings consistent with sarcoidosis, the extremely high sIL-2R level prevented complete exclusion of an associated lymphoproliferative process (Table 1).　

**Table 1:** Baseline laboratory, immunohematological, immunological, genetic, and coagulation findings at presentation This table summarizes bone marrow differential counts and cytogenetic findings, followed by splenic genetic and flow cytometric analyses performed at presentation. PNH - paroxysmal nocturnal hemoglobinuria; ACNA - antineutrophil cytoplasmic antibodies; INR -International Normalized Ratio

Parameter	Value	Reference range / Interpretation
Hematological findings		
White blood cell count	3900 /μL	3300-8600 /μL
Segmented neutrophils	33%	45-60%
Lymphocytes	19%	25-45%
Monocytes	17%	4-7%
Eosinophils	30%	1-4%
Basophils	1%	0-1%
Red blood cell count	1.99 × 10⁶ /μL	3.86-4.92 × 10⁶ /μL
Hemoglobin	6.4 g/dL	11.6-14.8 g/dL
Hematocrit	18.30%	35.1-44.4%
Mean corpuscular volume	92 fL	83.6-98.2 fL
Platelet count	6.2 × 10⁴ /μL	15.8-34.8 × 10⁴ /μL
Reticulocyte count	1.8 × 10⁴ /μL	1.9-13.2 × 10⁴ /μL
Immature platelet fraction	15.30%	1.0-7.0%
Biochemical findings		
Total protein	5.6 g/dL	6.6-8.1 g/dL
Albumin	3.6 g/dL	4.1-5.1 g/dL
Aspartate aminotransferase	21 U/L	13-30 U/L
Alanine aminotransferase	14 U/L	7-23 U/L
Alkaline phosphatase	71 U/L	38-113 U/L
γ-Glutamyl transpeptidase	24 U/L	9-32 U/L
Cholinesterase	189 U/L	240-486 U/L
Lactate dehydrogenase	416 U/L	124-222 U/L
Total bilirubin	1.5 mg/dL	0.4-1.5 mg/dL
Indirect bilirubin	1.0 mg/dL	0.3-1.0 mg/dL
Blood urea nitrogen	17.6 mg/dL	8-20 mg/dL
Creatinine	1.31 mg/dL	0.46-0.79 mg/dL
Uric acid	8.3 mg/dL	2.6-5.5 mg/dL
Total cholesterol	132 mg/dL	142-248 mg/dL
Triglycerides	221 mg/dL	40-234 mg/dL
Glucose	98 mg/dL	73-109 mg/dL
Sodium	134 mEq/L	138-145 mEq/L
Potassium	4.1 mEq/L	3.6-4.8 mEq/L
Chloride	100 mEq/L	101-108 mEq/L
Calcium	9.4 mg/dL	8.6-10.1 mg/dL
C-reactive protein	3.77 mg/dL	0.00-0.14 mg/dL
Iron	72 μg/dL	80-200 μg/dL
Unsaturated iron-binding capacity	182 μg/dL	168-252 μg/dL
Ferritin	441.6 ng/mL	39.4-340 ng/mL
Lysozyme	40 μg/mL	5.0-10.2 μg/mL
Angiotensin-converting enzyme	45.9 U/L	8.3-21.4 U/L
Urinalysis		
Urine protein	±	Negative
Urine glucose	Negative	Negative
Urine occult blood	Negative	Negative
Immunohematological findings		
Irregular antibodies	Anti-E antibody	Negative
Direct Coombs test	Negative	Negative
Indirect Coombs test	Negative	Negative
Soluble interleukin-2 receptor	26,600 U/mL	157-474 U/mL
PNH-type cells (granulocytes)	Type III: 0.000%	≥0.003%
PNH-type cells (granulocytes)	Type II + III: 0.001%	≥0.003%
Immunological and serological findings		
Complement component 3	94 mg/dL	86-160 mg/dL
Complement component 4	33 mg/dL	17-45 mg/dL
Immunoglobulin G	782 mg/dL	861-1747 mg/dL
Immunoglobulin A	114 mg/dL	93-393 mg/dL
Immunoglobulin M	16 mg/dL	33-183 mg/dL
Haptoglobin	<10 mg/dL	19-170 mg/dL
Erythropoietin	169 mIU/mL	4.2-23.7 mIU/mL
Antinuclear antibody	40	<40
Proteinase 3-ANCA	<1.0 U/mL	<3.5 U/mL
Myeloperoxidase-ANCA	<1.0 U/mL	<3.5 U/mL
Hepatitis B surface antigen	Negative	Negative
Hepatitis C virus antibody	Negative	Negative
Genetic analyses		
WT1 mRNA quantification	2.3 × 10² copy/μg RNA	<50 copy/μg RNA
JAK2 V617F mutation	Negative	Negative
Major BCR-ABL1 mRNA	Negative	Negative
Coagulation and fibrinolysis tests		
Prothrombin time-INR	1.29	0.85-1.15
Activated partial thromboplastin time	34s	24-34s
Fibrinogen	224.7 mg/dL	180-350 mg/dL
Fibrin/fibrinogen degradation products	17.9 μg/mL	<5.0 μg/mL
D-dimer	7.4 μg/mL	<1.0 μg/mL

**Figure 2:**
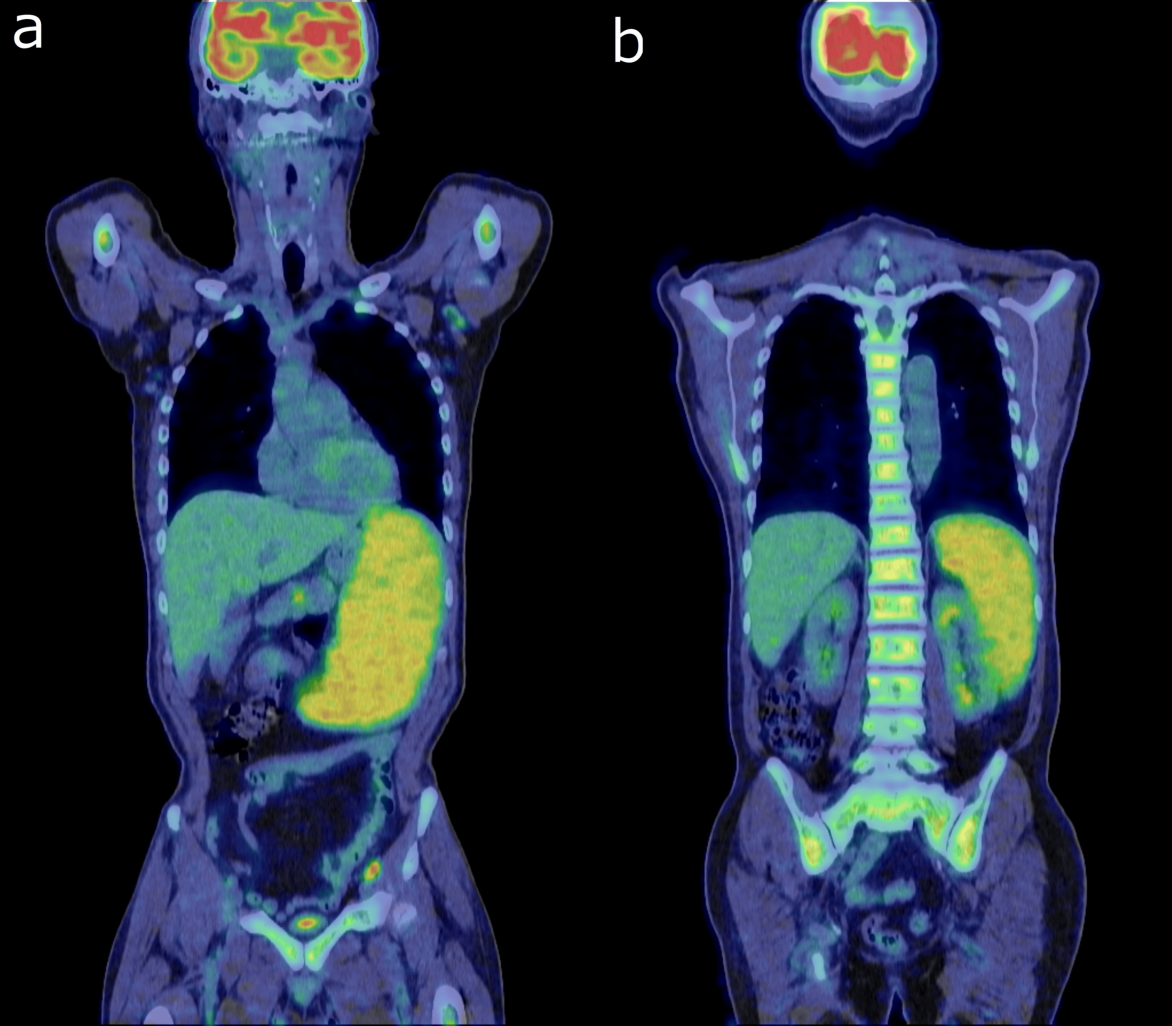
18F-FDG PET-CT 18F-fluorodeoxyglucose positron emission tomography-computed tomography (18F-FDG PET-CT) revealed intense fluorodeoxyglucose (FDG) uptake confined to the spleen (a) In addition, diffuse skeletal uptake was observed, suggestive of secondary bone marrow hyperactivity (b).

Fluorodeoxyglucose positron emission tomography-computed tomography (18F-FDG PET-CT) demonstrated intense FDG uptake in the spleen (Figure 2a), along with diffuse skeletal uptake suggestive of secondary bone marrow hyperactivity (Figure 2b).

Bone marrow examination revealed an increased number of megakaryocytes, and the immature platelet fraction% (IPF%) was elevated, indicating preserved platelet production (Table 2).

**Table 2:** Bone marrow and splenic pathological, genetic, and flow cytometric findings This table summarizes bone marrow differential counts and cytogenetic findings, followed by splenic genetic and flow cytometric analyses performed at presentation.

Parameter	Value	Reference range / interpretation
Bone marrow findings		
Nucleated cell count	35.0 × 10⁴ /μL	10.0-25.0 × 10⁴ /μL
Megakaryocytes	450 /μL	50-150 /μL
Proerythroblasts	0.80%	0.2-1.3%
Basophilic erythroblasts	5.20%	0.5-2.4%
Polychromatic erythroblasts	30.80%	17.9-29.2%
Orthochromatic erythroblasts	1.20%	0.4-4.6%
Myeloblasts	2.40%	0.2-1.5%
Promyelocytes	2.60%	2.3-4.1%
Myelocytes	9.80%	8.2-15.7%
Metamyelocytes	7.40%	9.6-24.6%
Band neutrophils	7.40%	9.5-15.3%
Segmented neutrophils	5.20%	6-12%
Eosinophils	12.00%	1.2-5.3%
Basophils	0.60%	0-0.2%
Lymphocytes	11.80%	11.1-23.2%
Monocytes	1.80%	0-0.8%
Plasma cells	1.00%	0.4-3.9%
Myeloid/erythroid ratio	1.25	1.5-3.3
Karyotype	46, XY	Normal male
Splenic genetic and flow cytometric analyses		
IgH gene rearrangement (JH region)	Negative	No clonal IgH rearrangement detected
T-cell receptor β (TCRβ) gene rearrangement (Cβ1 region)	Negative	No clonal TCRβ rearrangement detected
T-cell receptor γ (TCRγ) gene rearrangement (Jγ region)	Negative	No clonal TCRγ rearrangement detected
T-cell receptor δ (TCRδ) gene rearrangement (Jδ1 region)	Negative	No clonal TCRδ rearrangement detected
Flow cytometry (CD45 gating)	Normal	No abnormal lymphoid population detected
Karyotype	46, XY	Normal male

In addition, laboratory findings, including mildly elevated WT1 mRNA levels and reduced immunoglobulin levels (IgG 782 mg/dL, IgA 114 mg/dL, IgM 16 mg/dL), were carefully evaluated in the differential diagnosis.

Given the coexistence of hemolytic anemia and thrombocytopenia, further evaluation of the underlying mechanisms was required. Although reduced haptoglobin levels and elevated lactate dehydrogenase (LDH) suggested hemolysis, both direct and indirect Coombs tests were negative. The markedly elevated immature platelet fraction (IPF, 15%) and increased megakaryocytes in the bone marrow indicated preserved platelet production, suggesting secondary immune thrombocytopenia rather than bone marrow failure.

At that time, marked splenomegaly was present, and it remained unclear whether the splenic enlargement was attributable to sarcoidosis or an underlying lymphoproliferative disorder. Histopathological evaluation of the spleen was therefore considered essential for definitive diagnosis; however, splenectomy was initially deemed high risk due to severe thrombocytopenia.

Although immune-mediated hemolytic anemia could not be definitively confirmed, low-dose prednisolone therapy was initiated as a therapeutic and diagnostic trial for suspected secondary immune thrombocytopenia. A reduced dose of 20 mg/day was selected to minimize potential alteration of splenic histopathology prior to splenectomy. To secure a platelet count sufficient for surgical intervention, a thrombopoietin receptor agonist was added, allowing limitation of steroid exposure while achieving adequate platelet recovery.

Prednisolone was not initiated as standard therapy for active sarcoidosis, but rather as a low-dose diagnostic and therapeutic trial for suspected secondary immune thrombocytopenia.

Oral prednisolone at 20 mg/day was initiated, resulting in a gradual increase in the platelet count. After the addition of a thrombopoietin receptor agonist (TPO-RA), the platelet count rapidly increased to over 100,000/μL, making splenectomy feasible. In parallel, hemolytic anemia improved, and the sIL-2R level showed a marked decline even before surgery. Importantly, this rapid decline in sIL-2R prior to splenectomy argued against malignant lymphoma and supported an inflammatory etiology. The post-treatment serum sIL-2R level was 5,940 U/mL, measured 26 days after initiation of prednisolone therapy and two days prior to splenectomy. 

Because thrombopoietin receptor agonists are associated with an increased risk of thrombosis, particularly portal vein thrombosis (PVT) in patients undergoing splenectomy, thrombotic risk was carefully assessed before and during treatment. Contrast-enhanced computed tomography from the liver to the pelvis was performed on November 26, 2024, when the platelet count was 6.5 × 10⁴/μL, and again on December 26, 2024, when the platelet count peaked at 52.9 × 10⁴/μL; no evidence of PVT was detected on either examination. Given the severe thrombocytopenia at presentation and the high bleeding risk associated with splenectomy, prophylactic anticoagulation was not initiated, and close radiologic surveillance was prioritized. No thrombotic complications occurred throughout the clinical course.

**Figure 3:**
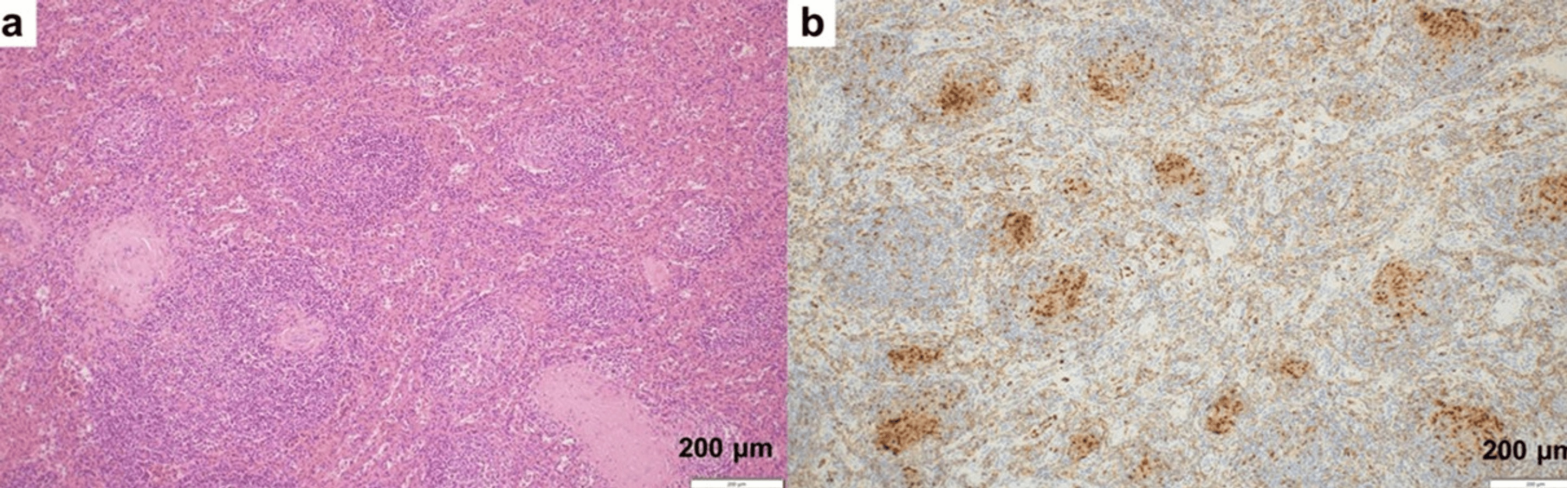
Histopathological findings of the spleen (a) Hematoxylin and eosin staining (×100): Numerous noncaseating granulomas are diffusely distributed. There is no evidence of malignancy, including lymphoma. (b) CD68 staining (x100): Positive staining corresponding to granulomas.

The resected spleen measured 15 × 12 × 5 cm and showed marked enlargement. On gross examination, the cut surface was homogeneous without identifiable nodules or mass lesions. Histopathological examination of the resected spleen revealed multiple non-caseating granulomas with focal necrosis (Figure 3) and no evidence of malignant lymphoma, leading to a diagnosis of splenic sarcoidosis.

**Figure 4:**
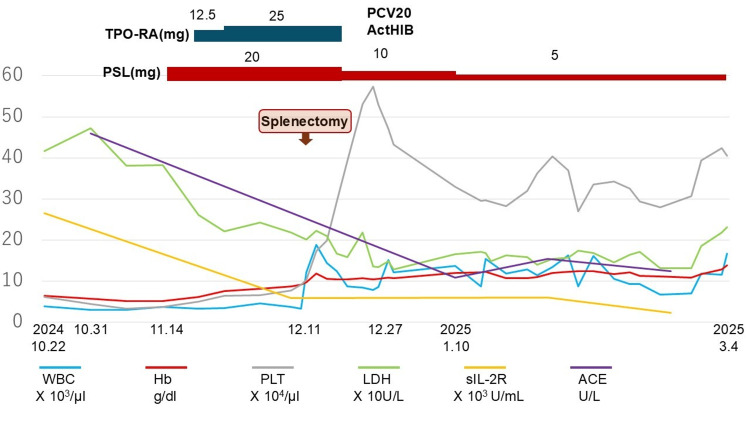
Clinical course of the patient's conditions and treatment Hb - hemoglobin; WBC - white blood cells; PLT - platelet count; LDH - lactate dehydrogenase; sIL-2R - soluble interleukin-2 receptor; ACE - angiotensin-converting enzyme; TPO-RA - thrombopoietin receptor agonist (eltrombopag); PSL - prednisolone; PCV2 -, 20-valent pneumococcal conjugate vaccine; ActHib - *Haemophilus influenzae *type b conjugate vaccine

After splenectomy and tapering of prednisolone, there was no recurrence of anemia or thrombocytopenia during follow-up (Figure 4).

Following splenectomy in December 2024, serum sIL-2R and ACE levels initially decreased (sIL-2R 2,250 U/mL and ACE 12.5 U/L on February 21, 2025), suggesting a transient response. However, both markers re-elevated by April 2025, requiring escalation of prednisolone therapy. Although temporary hematologic improvement was observed, disease activity subsequently fluctuated with recurrent thrombocytopenia and progressive anemia. Methotrexate, introduced as a steroid-sparing agent, was discontinued due to hematologic deterioration. Given persistent disease activity, azathioprine (100 mg/day) combined with low-dose prednisolone (5 mg/day) was initiated in October 2025, resulting in marked biochemical and hematologic improvement. As of December 25, 2025, sIL-2R and ACE levels had decreased to 7,190 U/mL and 13.9 U/L, respectively, with recovery of hemoglobin (8.4 g/dL) and platelet count (340×10³/µL). The patient has since remained clinically stable on maintenance therapy with azathioprine and low-dose prednisolone. 

## Discussion

Sarcoidosis is a systemic granulomatous disease that most commonly affects the lungs and lymph nodes. While splenic involvement has been reported in approximately 10-40% of patients with sarcoidosis, isolated splenic sarcoidosis without pulmonary or other organ involvement is exceedingly rare [5, 6]. In this case, WT1 mRNA was mildly elevated (230 copies). Although WT1 mRNA is a useful marker for myelodysplastic syndromes (MDS) and acute leukemia, the observed level was far below those typically reported in these conditions. Moreover, bone marrow morphology showed no dysplastic features, blast excess, or clonal hematologic abnormalities. Therefore, MDS was considered unlikely despite the presence of thrombocytopenia.

Bone marrow involvement in sarcoidosis is reported in fewer than 10% of cases, usually as part of systemic disease [4,7]. Cases lacking hilar or mediastinal lymphadenopathy and involving only the spleen or bone marrow are classified as limited-form extrapulmonary sarcoidosis [8].

Although isolated involvement of either the spleen or bone marrow has been previously reported, to our knowledge, this appears to be the first reported case of sarcoidosis confined to both organs simultaneously. Bone marrow sarcoidosis is extremely rare and typically occurs in the context of multisystem disease. In most previously reported cases, splenectomy led to improvements in clinical symptoms and hematologic abnormalities, suggesting that splenic involvement may represent the primary disease process, while bone marrow findings are often considered secondary immune responses or extensions of inflammation [9]. In our case, non-caseating epithelioid granulomas, characteristic of sarcoidosis, were observed in both the spleen and bone marrow, with greater density and distribution noted in the marrow. Moreover, 18F-FDG PET-CT showed intense uptake not only in the spleen but also diffusely in the bone marrow, including the contralateral iliac bone, suggesting that the bone marrow may have represented the primary site of disease [10, 11]. The spleen plays a physiological role in extramedullary hematopoiesis and immune regulation. In the setting of bone marrow dysfunction or systemic inflammation, reactive changes in splenic architecture and function are frequently observed [12]. However, since the patient had already received prednisolone therapy prior to splenectomy, it is possible that granulomatous lesions in the spleen had regressed compared to those in the marrow, making it difficult to definitively identify the primary lesion.

Sarcoidosis is characterized by Th1-dominant immune activation with increased production of interleukin-2, which is reflected by elevated serum soluble interleukin-2 receptor levels and correlates with disease activity [13, 14]. Such an immune imbalance has been reported to cause relative suppression of B-cell function, potentially leading to reduced immunoglobulin production in some patients with active sarcoidosis [15]. In the present case, the decreased levels of IgG, IgA, and IgM were therefore considered to reflect immune dysregulation associated with active sarcoidosis rather than lymphoma-related humoral immunodeficiency. In addition, hypersplenism may have contributed to the reduction in circulating immunoglobulins.

This case also presented with Coombs-negative hemolytic anemia and thrombocytopenia. The observed increase in megakaryocytes within the marrow and the elevated immature platelet fraction (IPF%) suggested that the thrombocytopenia was primarily due to hypersplenism caused by massive splenomegaly. However, sarcoidosis-associated immune thrombocytopenia (ITP) has also been reported [16], making a clear distinction between these mechanisms challenging. Splenectomy was necessary for definitive diagnosis, but an increase in the platelet count to approximately 100,000/μL was required for surgery. Although steroid therapy can cause regression of sarcoid lesions and potentially hinder histologic diagnosis, we judged that complete disappearance of the lesions was unlikely and initiated prednisolone at 20 mg/day. Due to a lack of platelet response, a thrombopoietin receptor agonist (TPO-RA) was added to the regimen, resulting in a rapid increase in the platelet count to the target level. Although off-label for hypersplenism, TPO-RAs have shown efficacy in such contexts [17].

In retrospect, although low-dose prednisolone was selected to preserve splenic histopathology and reduce treatment-related risks, a higher initial corticosteroid dose might have resulted in a more rapid hematologic response, particularly with respect to hemolytic anemia.

However, given the diagnostic uncertainty at presentation and the need to obtain an unmodified splenic specimen, the chosen strategy allowed effective platelet recovery with minimal steroid exposure when combined with a thrombopoietin receptor agonist. This case highlights the importance of individualized corticosteroid dosing, balancing diagnostic priorities and therapeutic efficacy in similar clinical settings.

Reports of hemolytic anemia in sarcoidosis are rare and typically involve Coombs-positive cases [3]. To our knowledge, there have been no prior reports of Coombs-negative hemolytic anemia associated with sarcoidosis, highlighting the uniqueness of this case. While the pathophysiology of Coombs-negative hemolytic anemia remains unclear, several possible mechanisms have been proposed: complement dysregulation leading to antibody-independent red cell destruction; the presence of low-affinity autoantibodies undetectable by conventional Coombs testing; and increased red cell destruction due to hypersplenism.

In our patient, immune dysregulation and granulomatous infiltration associated with sarcoidosis may have contributed to these mechanisms. Notably, hemolytic anemia improved markedly following prednisolone therapy, suggesting the likely involvement of low-affinity autoantibodies.

Patients with sarcoidosis are at increased risk of developing lymphomas, including both B- and T-cell types, typically within two to eight years after onset. Brincker first described this phenomenon in 1974 and later coined the term sarcoidosis-lymphoma syndrome in 1986 [18, 19]. More recent reviews and clinical studies have further supported this association and provided updated perspectives on its epidemiology, clinical features, and underlying pathogenetic mechanisms [20-22].

Sarcoidosis is characterized by the activation of CD4-positive T cells, primarily Th1 cells, which secrete cytokines such as IL-2 that perpetuate immune activation. Serum soluble interleukin-2 receptor (sIL-2R) levels are considered a useful marker of disease activity and are elevated in many cases [2, 23]. In previous reports, sIL-2R levels in sarcoidosis rarely exceeded 6100 U/mL [2, 24]. In contrast, our patient showed an unusually high level of 26,600 U/mL at presentation. Although this decreased to 5940 U/mL following prednisolone therapy, the possibility of underlying lymphoma could not be definitively ruled out until histologic examination of the spleen excluded its presence.

It should be emphasized that even extremely elevated serum sIL-2R levels are not pathognomonic for malignant lymphoma and cannot establish a definitive diagnosis in the absence of histopathological confirmation. Although markedly increased sIL-2R levels often raise a strong clinical suspicion for lymphoproliferative disorders, sIL-2R fundamentally reflects T-cell activation and may reach very high levels in inflammatory granulomatous diseases such as sarcoidosis.

Importantly, this case serves as a cautionary example against prematurely discontinuing diagnostic evaluation or initiating empirical chemotherapy in patients presenting with massive splenomegaly and markedly elevated sIL-2R levels. Notably, the rapid decline in sIL-2R observed after the initiation of corticosteroid therapy, prior to splenectomy, represented a key differentiating feature favoring an inflammatory granulomatous process over malignant lymphoma. This dynamic change in sIL-2R levels constitutes an important clinical pearl and may aid clinicians in avoiding misdiagnosis and unnecessary cytotoxic treatment in similar clinical settings.

Prednisolone was subsequently tapered to a maintenance dose without recurrence of anemia or thrombocytopenia, supporting the interpretation that immune dysregulation associated with sarcoidosis was the primary driver of the hematologic abnormalities in this case.

## Conclusions

This case provides novel insights into the mechanisms underlying Coombs-negative hemolytic anemia and thrombocytopenia in sarcoidosis, and highlights the diagnostic and therapeutic value of splenectomy. Furthermore, it represents the first documented case of sarcoidosis limited to both the spleen and bone marrow, contributing to a deeper clinical and pathophysiological understanding of this rare disease presentation.
